# A Stable and Indurative Superhydrophobic Film with Excellent Anti-Bioadhesive Performance for 6061 Al Protection

**DOI:** 10.3390/ma13235564

**Published:** 2020-12-06

**Authors:** Jie Liu, Xinwen Zhang, Ruoyun Wang, Fei Long, Lei Liu

**Affiliations:** 1 State Key Laboratory of Metal Matrix Composites, School of Material Science and Engineering, Shanghai Jiao Tong University, Shanghai 200240, China; lindialj@sjtu.edu.cn (J.L.); zhangxw@sjtu.edu.cn (X.Z.); microarc@sjtu.edu.cn (R.W.); longfeimse@sjtu.edu.cn (F.L.); 2 Collaborative Innovation Center for Advanced Ship and Deep-Sea Exploration, Shanghai Jiao Tong University, Shanghai 200240, China

**Keywords:** organic-inorganic composite, superhydrophobicity, mechanical robustness, anti-bioadhesive property

## Abstract

Superhydrophobic surfaces have attracted intensive attention in the antifouling field because of their excellent anti-bioadhesive performance and environmental friendliness. However, promising surfaces have met great challenges of poor mechanical robustness under harsh serving conditions. Herein, an organic-inorganic composite strategy, that the silane-modified TiO_2_ nanoparticles are compounded into the porous framework provided by the stable and indurative aluminum oxide film, is proposed to address the common serious problem in superhydrophobic surfaces. Different from the traditional superhydrophobic surfaces, this composite film possesses a ~18 μm thick layer which can provide strong support to silane-modified TiO_2_ nanoparticles. The resulting film can reserve superhydrophobicity to the surface even after a thickness loss of ~15 μm under continuous abrasion. At the same time, the results of the bacterial adhesive tests also verify that the film has the same long-term anti-bioadhesive performance. The film with superhydrophobicity, excellent anti-bioadhesive property, and stable robustness will make it a promising candidate for serving in a harsh environment, and the design concept of this film could be applied to various substrates.

## 1. Introduction

Anti-bioadhesive surfaces have become research hotpots because of the urgent need for seeking the alternative to replace the traditional antibacterial coatings that always contain fungicides [1,2]. Numerous researchers [3,4] have done much investigation on the strategies to develop environmentally friendly anti-bioadhesive surfaces, among which, superhydrophobic surfaces with high water contact angle (WCA, ≥150°) have shown super anti-bioadhesive performance and nontoxicity to environments. To achieve the superhydrophobic surfaces, a common approach is modifying the surface with the low-surface-energy chemistry as well as decreasing the contact area between liquid and solid via creating the micro-/nanoscale textures or fabricating the nanoporous structures [5,6,7]. To increase the surface hydrophobic property, many methods have been reported, including etching [8], electrochemical deposition [9], magnetron sputtering [10], lithographing [11], a thermochemical synthetic method [12], and sol–gel processing [13]. For example, Maharana et al. fabricate a nano-hierarchical structured Cu-ZrO_2_ nano-cone arrays to acquire hydrophobicity [14]. These researches indicate that the effects of the microstructure geometries, the high aspect ratio guard ring structure, and the hierarchical surface roughness are the key factors that can improve the superhydrophobicity. Whereas, a small fraction of the overall area in contact with liquid results in high local pressure on the contact area, which weakens the surface robustness against abrasion [5,6,15]. 

To improve the mechanical robustness of the superhydrophobic coatings, various approaches have been explored, such as enhancing the bonding force between the coating and the substrate [16], strengthening the hardness of the coating [17], fabricating a biomimetic self-healing surface [18,19,20], and creating a self-similar structure by the low-output methods, such as lithographing and magnetron sputtering [10,11,21,22]. Among these approaches, coatings with high hardness and strong bonding force have shown improved robustness comparing with the general coatings. For instance, Du et al. [23] have reported a super-robust hydrophobic coating fabricated by multi-arc ion plating, and the coating exhibits enhanced hardness, good adhesive force, and excellent wear resistance, whereas this strategy has resulted in only modest advancements in mechanical robustness and it will cause rapid failure once the coating suffers damage [6]. For the bionic strategy, it is often too hard and expensive to mimic the intrinsically self-repairing ability [5]. Therefore, it is urgently needed to develop a robust superhydrophobic coating that not only possesses high hardness, strong bonding force, and large self-similar layer but also can be mass-produced at a low cost. 

Aluminum oxide film [24,25] with high hardness and good anti-corrosion property, has widely used as the surface protective layer of the structural material applied in ships, aircraft, and vehicles, and so on. The nanoporous microstructures of the aluminum oxide film can be controlled by adjusting the fabricating parameters [26], and the selected nanoporous surfaces can act as the container to load the functional nanofiller. Many powders—such as TiO_2_ [27], Cu_2_O [28], zirconia [29,30,31], silica [32], ZTA [33], and SiC [34]—have reported as the functional powders to modify the metallic or ceramic surfaces, and these powders are obtained by various methods including sol–gel [35], coprecipitation [36], subcritical drying [37], and so on. For instance, M.Taha et al. have fabricated a SiC-reinforced 6061 Al to achieve the physical, mechanical, and electrical properties [34]. Among these powders, TiO_2_ nanoparticles, with good dispersibility, will provide promising applications in surface engineering since they can effectively increase the surface roughness and improve the specific surface area [27]. Moreover, the nanoparticles enable the formation of the nanocomposite induced by the low-surface-energy organic modifiers [38]. Perfluorodecyltrimethoxysilane (PFDS), with the lowest surface energy and the best wetting-repellency, is a commercial surface-active agent widely used in the coatings of electronic screens [39]. However, the adhesive force between the substrate and the PFDS-contained coating is just through the van der Waals force in the traditional applications. Herein, we suggest combining the TiO_2_ nanoparticles with PFDS to form a nanoparticle-induced sol–gel, and then filling the sol–gel into the nanopores of the aluminum oxide film. Thus, the active hydrophobic groups will be transplanted to the nanoporous surface through the strong chemical bonds between pore-walls and active groups [40].

In this work, we design a superhydrophobic surface with excellent mechanical robustness against abrasion that is achieved via a simple process. The microstructure of the surface contains a thick nanoporous framework filled with functional nanoparticles to provide water repellency and durability. The choice of the nanoporous frameworks with different microstructure geometries has been discussed based on the Cassie–Baxter theory. As an application, the anti-bioadhesive performance of the surface before and after abrasion has been investigated. The research sense of this work is to provide an alternative strategy of fabricating an excellent robust superhydrophobic surface in an affordable manufacturing method and to launch a design concept that can be conveniently applied to the other metal surfaces.

## 2. Materials and Experiments

### 2.1. Materials

6061 aluminum alloy plate (6061 Al, 5.0 cm × 5.0 cm) was selected as the substrate of the fabricated coating. Phosphoric acid (H_3_PO_4_, 15 wt%), sulfuric acid (H_2_SO_4_, 200 g/L), oxalic acid (OA, 5 wt%) were the electrolytes used in the anodizing process. The nanofiller (PFDS/TiO_2_) was the mixture of 1 g 1H, 1H, 2H, 2H-perfluorodecyltrimethoxysilane (PFDS, C_13_H_13_F_17_O_3_Si), 98 g ethyl alcohol, and 2 g TiO_2_ nanoparticles (The optimized amount of TiO_2_ nanoparticles was decided after the testing campaign, and the influence of the TiO_2_ amount to the microstructures was discussed in Figure S4 in Supporting Information). *Escherichia coli* (*E. coli*) and *Staphylococcus aureus* (*S. aureus*) were used in the anti-bioadhesive testing. The phosphate buffer solution (PBS) compounded with 1.36 g/L KH_2_PO_4_, 2.83 g/L Na_2_HPO_4_, and 0.8 g/L NaCl; the fluid nutrient medium (FNM) composed of 5 g/L NaCl, 5 g/L yeast extract, and 10 g/L peptone; and the solid nutrient medium (SNM) with the chemical composition of 5 g/L NaCl, 5 g/L yeast extract, 10 g/L peptone, and 20 g/L agar. All the chemical and biological reagents were purchased from Beijing Innochem Technology Co., Ltd. (Beijing, China).

### 2.2. Fabrication of the Anti-Bioadhesive Coating

**Figure 1:**
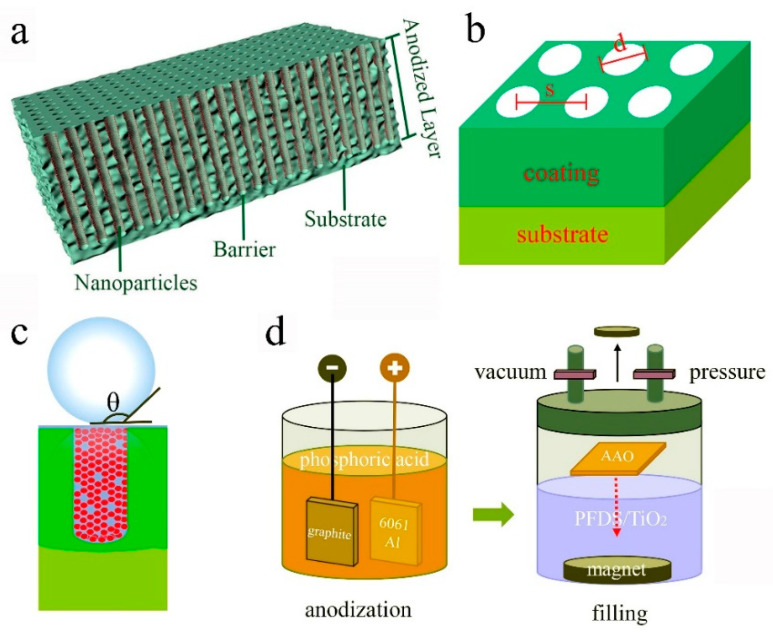
Schematic illustration of (**a**) the superhydrophobic surface, (**b**) the coating geometric model, (**c**) Cassie–Baxter model, (**d**) the schematic of fabricating the superhydrophobic surface.

Aluminum oxide film was prepared by anodizing 6061 Al using a two-electrode system as the schematic in Figure 1d. The 6061 Al plate was as an anode electrode, and the graphite was as the cathode electrode. The anodizing process was performed in a thermostat water bath. The regulated power source (NF KP3000GS, Tokyo, Japan) was used to supply constant voltage. To select an optimized anodized film, three electrolytes (H_3_PO_4_, H_2_SO_4_, and OA) were tried in the anodizing process, and the detailed parameters for each electrolyte were listed in Table 1. Then, the optimized aluminum oxide film was immersed in the prepared PFDS/TiO_2_. The immersed aluminum oxide film was placed in a sealed chamber with a vacuum environment of 10^−1^ Pa for 2 h. Then, the seal chamber was filled with N_2_ to the pressure of 10^6^ Pa and kept for another 2 h. Finally, the full filled nanoporous surface with the self-similar structure was formed on 6061 Al. 

### 2.3. Chemical and Morphological Characterization

The morphology of the surface/cross-section and the distribution of the elements were characterized by the scanning electron microscope (SEM, JEOL 7600F, Tokyo, Japan) using a tungsten filament under an acceleration voltage of 15 keV. To enhance the surface conductivity, the SEM samples were sputtered with Pt using a turbomolecular pumped sputter coater (Quorum Q 150T ES plus, East Sussex, UK) at a current of 15 mA for 20 s. The time of flight secondary ion mass spectrometry (TOF-SIMS, TESCAN GAIA3, Brno, Czech) was employed to quantificationally detect all of the surface elements including C, O, and H using the gallium ion source. In the TOF-SIMS experiment, an accelerating potential of 30 keV was used to optimize for spatial resolution (~1 m, with a corresponding mass resolution of ~3,000 m/ m). Data acquisition was set for a mass range of 0 to 500 atomic mass units. The spectral resolution was sufficient to uniquely identify elemental signals, which was corroborated by their isotopic contributions where possible. Charge build-up was counteracted by the use of interleaved pulsed electron neutralization (60 V extraction, 10 V bias). Spectra were acquired in both negative and positive ion mode. The phase composition was determined by X-ray diffraction (XRD, Bruker D8 ADVANCE, Leipzig, Germany) with a Cu target (λ = 0.154056 nm) under the working voltage of 40 kV and current of 30 mA, and the data were collected at 2θ = 10–90° at a scanning rate of 2θ = 2°/min. Fourier transform infrared spectrum (FTIR, Bruker EQUINOX 55, Leipzig, Germany) was recorded in the range from 500 to 4000 cm^−1^ to detect the organic groups in PFDS. The elemental bonding and composition were acquired by X-ray photoelectron spectroscopy (XPS, Kratos AXIS Ultra DLD, Manchester, UK) at a take-off angle of 90° using an X-ray source of Al Kα. The correct charging was calibrated by C 1s peak at 284.8 eV. Micro Vickers hardness instrument (Buehler, 1600-6406, Illinois, USA) was used to measure the hardness of the coating. The surface roughness was characterized by the atomic force microscope (AFM, Nanonavi E-Sweep, Tokyo, Japan).

### 2.4. Wettability and Robustness Testing

The liquid drop test was applied to measure the static water contact angle (WCA) by the video optical contact angle measuring instrument (Kruss, DSA100, Hamburg, Germany). To study the mechanical robustness of the superhydrophobic coating, the WCA of the surface after abrasion was tested. The surface abrasion was conducted via the cyclic linear motion of the emery paper (2000#) under the fixed weight (500 g), and the schematic illustration was shown in Supporting Information (Figure S1). All the samples after abrasion were kept the consistent roughness to ensure that the change of wettability was just caused by the thickness loss but the surface roughness. 

### 2.5. Anti-Bioadhesive Testing

The anti-bioadhesive properties against *Escherichia coli* (*E. coli*) and *Staphylococcus aureus* (*S. aureus*) were evaluated by the plate count method. Before the experiments, the solutions including PBS, fluid nutrient medium, solid nutrient medium, and the prepared samples were all sterilized at 121 °C for 30 min. Bacteria suspensions in the culture tubes, where the bacteria concentration was ~10^6^ colony forming units (CFU/mL), were incubated in a shaker with 150 rpm at 37 °C. After incubated for 12 h, the bacteria suspensions were taken out and diluted to 10%, that is, the bacteria concentration became ~10^5^ CFU/mL. Then, the sterilized samples were put into a 24-well cell culture plate and instill the diluted bacterial suspension (100 μL). The 24-well cell culture plate was kept in an incubator at 37 °C for 24 h. After that, the samples dipping in the bacteria suspensions were taken out and washed out with PBS. The cleaned samples were placed into tubes with PBS (1 mL) and put into the ultrasonic cleaning instrument to shocked off the adhesive bacteria on samples. The PBS with shocked-off bacteria were spread over the solid nutrient medium plates. The plates were kept in an incubator at 37 °C and taken out after 24 h later to count the bacterial colonies. The anti-bioadhesive performance was expressed by the inhibition ratio (IR%) that was calculated by the following equation [40], where CFU was the colony-forming units formed in the plate

(1) $$\mathrm{IR}\%=\frac{\mathrm{CFU}\;\mathrm{of}\;\mathrm{control}\;\mathrm{group}-\mathrm{CFU}\;\mathrm{of}\;\mathrm{experimental}\;\mathrm{group}}{\mathrm{CFU}\;\mathrm{of}\;\mathrm{control}\;\mathrm{group}}\times 100\%$$

### 2.6. Adhesion Test

The adhesion of the superhydrophobic layer was measured through a scratch test and bending test. The scratch test was performed under a loading rate of 30 N/m using a scratch tester (Anton Paar RST^3^, Shanghai, China), and the intensity of the sound signal would have saltation with the coating fracture. The bending test was done using a universal testing machine (Instron^®^ 3400, Boston, MA, USA). The bending sample was prepared to be 2 mm thick, 40 mm long, and 10 mm wide; and the testing span was set to be 32 mm. The acquisition data in the bending test were plotted as the curve of bending strength with the displacement.

## 3. Results and Discussion

### 3.1. Design Strategy to Obtain the Robust Superhydrophobic Coating

The strategy for achieving a robust superhydrophobic surface is to fabricate an organic–inorganic composite layer on 6061 Al through filling the aluminum oxide film with PFDS/TiO_2_, as shown in the schematic illustration in Figure 1a. aluminum oxide film, as the foundation of the framework, intrinsically has high hardness, strong bonding force with the substrate, corrosion resistance, and good processing property. Furthermore, the nanopores of the aluminum oxide film can be used as the storage of the hydrophobic functional groups. After being filled with the nanoparticle-induced low-surface-energy nanofiller, the surface with the nanoporous framework will become to repel water. The wetting state of the surface can be expressed by the Cassie–Baxter model (Figure 1c) that the droplet keeps above the Cassie interface [6,23] due to the existence of the air cushion in the nanoporous structures. According to Cassie–Baxter theory, the WCA of the superhydrophobic surface, θ, can be expressed by the Cassie–Baxter relation [6]

(2) $$\cos\theta_{\gamma }=f_{\mathrm{ls}}\times \left({\cos\theta }_{0}+1\right)-1$$

where, $\theta_{\gamma }$ is the apparent contact angle, $\theta_{0}$ is the intrinsic contact angle, $f_{\mathrm{ls}}$ is the percentage of the liquid-solid interface area. From the relation, it can get that $\theta_{\gamma }$ has a negative correlation with $f_{\mathrm{ls}}$ ($f_{\mathrm{ls}}<1$). Therefore, minimizing the liquid-solid interface can improve the WCA, but it will cause a decrease in mechanical robustness. 

**Figure 2:**
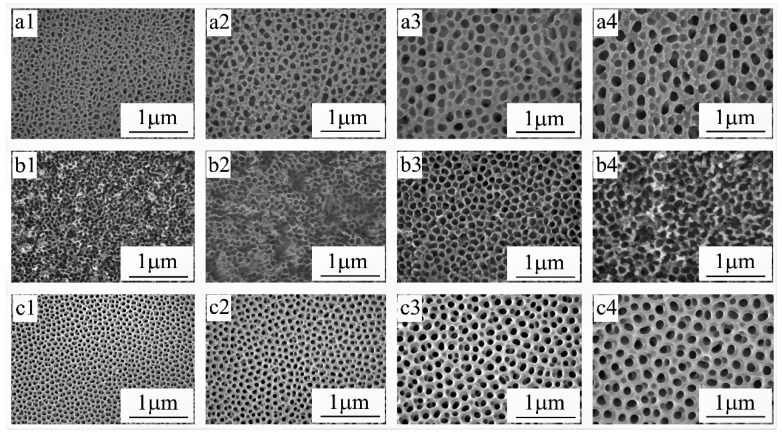
Aluminum oxide film obtained via the anodizing process in the electrolyte of (**a**) phosphoric acid with a concentration of 15 wt% under a constant voltage of 40 V (**a1**), 50 V (**a2**), 60 V (**a3**), and 70 V (**a4**); (**b**) sulfuric acid with a concentration of 11 wt% under a constant voltage of 20 V (**b1**), 25 V (**b2**), 30 V (**b3**), and 35 V (**b4**); (**c**) oxalic acid with a concentration of 5 wt% under a constant voltage of 40 V (**c1**), 45 V (**c2**), 50 V (**c3**), and 55 V (**c4**).

To optimize the robustness and superhydrophobicity, we prepare these aluminum oxide films in different electrolyte systems under various anodizing voltage. SEM images in Figure 2 show the morphology of aluminum oxide film formed in the three electrolytes under various anodizing voltage, and the corresponding average pore diameter, porosity (see the porosity calculation method in Figure S2), and hardness (see the testing details in Figure S3) are listed in Table 2. Results show that aluminum oxide film prepared in the H_3_PO_4_ electrolyte system (Figure 2a) has large nanopores (~130 nm), and the diameter of the nanopores increases with the anodizing voltage. However, the hardness of aluminum oxide film formed in the phosphoric acid system is below 140 HV, which is not very conducive to engineering application. Aluminum oxide film fabricated in the H_2_SO_4_ electrolyte system tends to form small nanopores (~85 nm) and thin pore-wall (Figure 2b), and the hardness is above 270 HV. Whereas, the small nanopores are not fit as the modifier storage which will be filled with nanoparticle-induced nanofiller. Figure 2c displays the morphology of the aluminum oxide film formed in the OA electrolyte that possesses large pores, and the hardness is relatively high. Therefore, the pore size of the fabricated aluminum oxide film in different electrolytes increases in the following order: H_2_SO_4_ < OA < H_3_PO_4_, and the hardness heightens in the following order: H_3_PO_4_ < H_2_SO_4_ < OA. Therefore, the optimized aluminum oxide film that simultaneously has a large pore size, high porosity, and high hardness can be achieved in the OA electrolyte system (Figure 2c). 

As shown in Figure 2c, the nanoporous aluminum oxide film formed in OA electrolyte exhibits fine roughness and a unique nanoporous structure with high uniformity. According to the above comprehensive analysis of the requirement in the filling process, the mechanical robustness, and the wetting model, we select the aluminum oxide film with a pore size of ~100 nm, the porosity of 45.9% (see the calculating details in Figure S2), and the hardness more than 350 HV (Table 2) as the metal framework of the coating. Three reasons are listed to explain the choice: first, the nanopore sized ~100 nm is large enough for the nanofiller with TiO_2_ nanoparticles sized ~15 nm; besides, based on the Cassie–Baxter relation, the theoretical WCA of the filled aluminum oxide film with the porosity of 45.9% can reach ~160° (see the calculating details in Supporting Information); moreover, the high hardness (above 350 HV) is sufficient to meet the requirements of the engineering application. Thus, the optimized aluminum oxide film has been picked out as the metal framework of the coating. Previous studies [30,41] have pointed to that a large diameter of dispersed TiO_2_ nanoparticles (sized ~15 nm) is recommended as the structural modifier to increase the surface roughness and improve the specific surface area. For both of the above reasons, an aluminum oxide film with ~100 nm diameter is selected as the nanoporous framework of the surface, and TiO_2_ nanoparticles with ~15 nm diameter are chosen as the nanofiller.

### 3.2. Analysis of the Morphologies and Components

**Figure 3:**
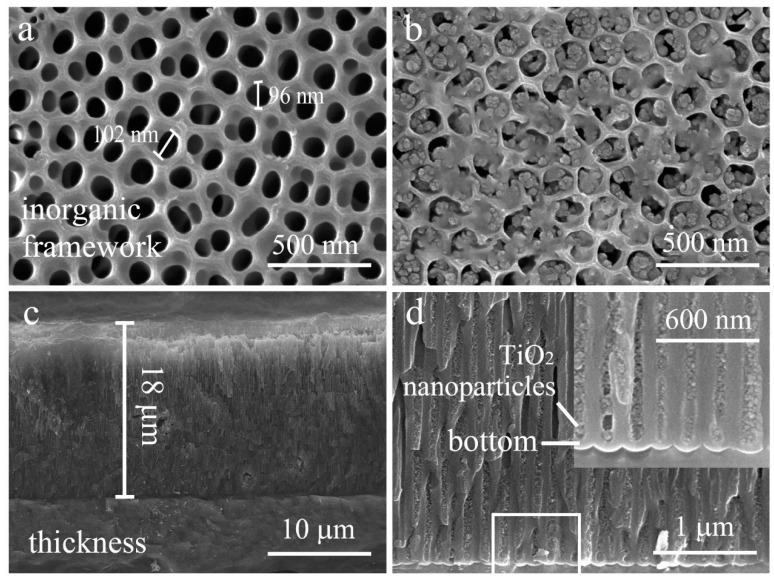
SEM images of (**a**) the AAO obtained via anodizing the 6061 Al under a constant voltage of 50 V in 5 wt% OA electrolyte system, (**b**) the coating surface with the nanoporous framework, (**c**) the coating cross-section with a thickness of 18 μm, and (**d**) the bottom full filled with nanoparticle-induced superhydrophobic nanofiller.

Morphologies of the surface and cross-section are characterized by SEM (Figure 3). Figure 3a displays the aluminum oxide film structure that is composed of nanopores (sized ~100 nm) and pore walls (sized ~35 nm), and the nanopores and walls are evenly spaced with no presence of collapses and cracks. To obtain the superhydrophobic surface, the aluminum oxide film is filled with low-surface-energy PFDS/TiO_2_. As shown in Figure 3b, the filled surface involves the random and evenly honeycomb-like structures, and the nanoparticles inside of the nanopores have an average diameter of 15 nm. To achieve a durable superhydrophobicity, the organic–inorganic framework is designed to form a honeycomb-like structure with a large thickness in the depth direction, that can endow the coating with robustness against abrasion by sacrificing the upper layers in a self-similar manner. Figure 3c displays the cross-section of the coating, and the length from the upper surface to the bottom is shown to be ~18 μm. In the magnified SEM image of the cross-section (Figure 3d), it can be seen that the nanoparticles are deep filled into the coating bottom. The fully filled coating at ~18 μm thick can provide the sustaining superhydrophobicity and excellent robustness under continuous abrasion. 

**Figure 4:**
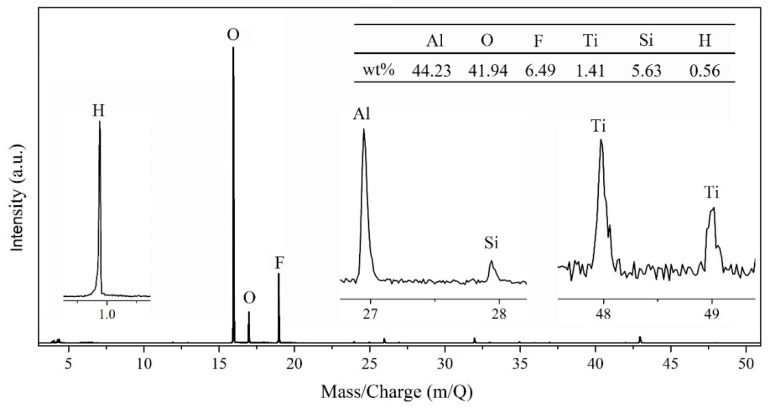
TOF-SIMS for quantificationally determining the elements including Al, Ti, Si, C, O, and H of the coating.

**Figure 5:**
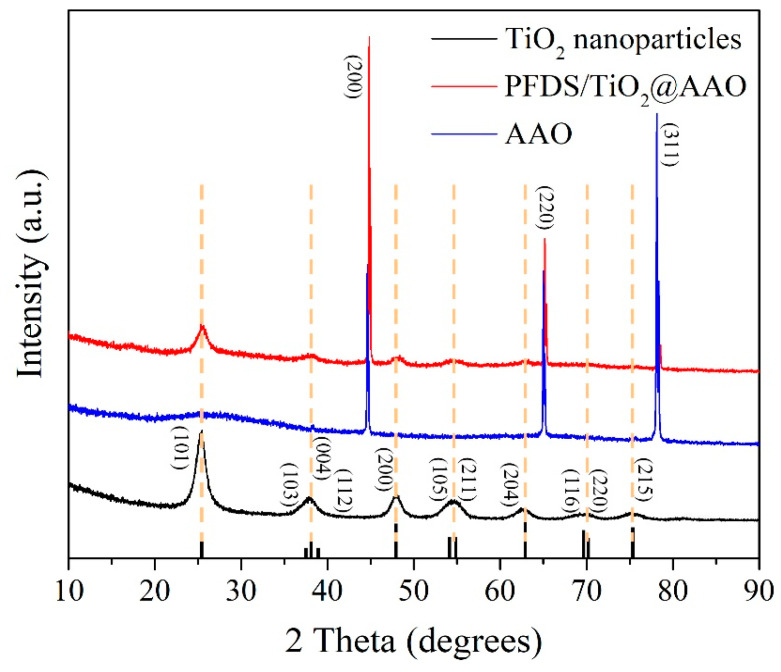
XRD patterns of the TiO_2_ nanoparticles, the AAO, and the fabricated PFDS/TiO_2_@AAO surface.

**Figure 6:**
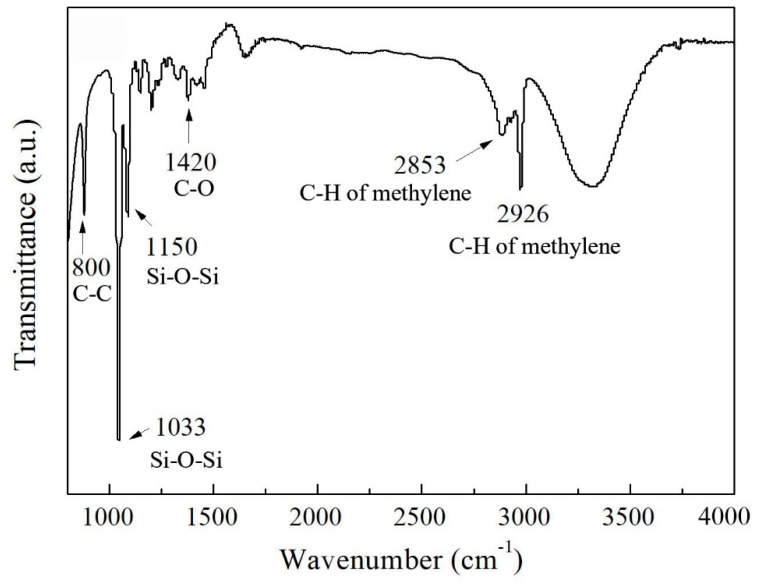
FTIR of the PFDS/TiO_2_@AAO, showing the enlarged view of the spectra in the range 500 cm^−1^ to 4000 cm^−1^.

**Figure 7:**
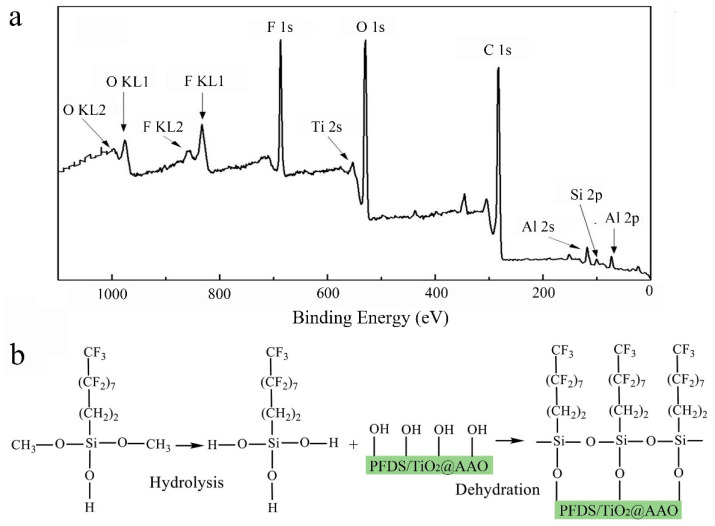
(**a**) XPS analysis of the superhydrophobic coating. (**b**) Schematic illustration of the functional mechanism of the PFDS in the superhydrophobic coating.

TOF-SIMS represents the elemental peaks on the coating, and the quantificational elemental contents of Al, Ti, Si, C, O, and H are listed in the insert table in Figure 4. Strictly speaking, a large amount of Al element attributes to the aluminum oxide film and the 6061 Al substrate, the existence of Ti is due to TiO_2_ nanoparticles, Si and C are the main elements of PFDS, and O element, with a high percentage, derives from TiO_2_, aluminum oxide film, and PFDS. To distinguish the elemental forms, XRD and FTIR are employed to characterize the metal composition and organic matter, respectively. Figure 5 shows the XRD pattern of the TiO_2_ nanoparticles, the AAO, and the fabricated PFDS/TiO_2_@AAO surface. The XRD of TiO_2_ nanoparticles reveals the presence of the anatase polymorph with the crystallographic planes in orientations of (101), (103), (004), (112), (200), (105), (211), (204), (116), (220), and (215) [30,42]. These crystallographic planes are consistent with those reported in the previous paper [22]. As the XRD result, AAO has a peak curve bread that represents amorphous and three crystallographic planes in orientations of (200), (220), and (311). These crystallographic planes of TiO_2_ nanoparticles have been found in the PFDS/TiO_2_@AAO surface, which indicates that it cannot cause the crystallographic-plane transformation of TiO_2_ nanoparticles in the modifying process. FTIR (Figure 6) records the organic groups in the coating. The absorption peaks appear at 1033 cm^−1^ and 1150 cm^−1^ (consistent with the Si-O-Si stretching), 1420 cm^−1^ (indicating the C-O stretching), 2853 cm^−1^, and 2926 cm^−1^ (referring to the C-H of methylene) [43]. This indicates that the functional organic groups are attached to the surface and endow the surface with the good water-repellent property. However, these results cannot illustrate the bonding form between the organic groups and the aluminum oxide film. Here, XPS is performed to investigate the chemical conditions of the superhydrophobic surface. Figure 7a shows that the elements of C, F, and Si are detected in the surface, implying that the surface has been covered with silane. Based on the previous research, it can be deduced that the chemical bonds of Al-O-Al in aluminum oxide film and the Si-O in PFDS are opened in the hydroxylation process, and the -OH groups (existing in ethyl alcohol) are grafted to these open chemical bonds [44,45]. Then, the hydroxylated aluminum oxide film and PFDS are chemically combined in the dehydration process [46,47] as the schematic shown in Figure 7b. Thus, the low-surface-energy organic groups are grafted on the framework of the coating through the two processes of hydroxylation and dehydration.

### 3.3. Superhydrophilicity, Anti-Bioadhesive Performance, and Robustness

**Figure 8:**
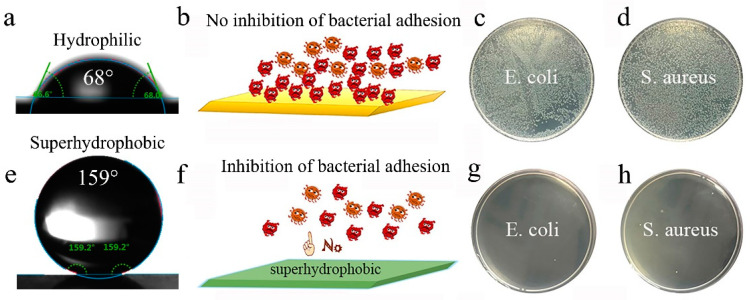
Water repellent performance and anti-adhesion behavior of the unmodified aluminum oxide film and the superhydrophobic PFDS/TiO_2_@AAO. (**a**) Water repellent performance of the unmodified aluminum oxide film. (**b**) Schematic illustration of the bacterial adhesion on the unmodified aluminum oxide film. (**c**,**d**) Bacterial adhesion testing of the unmodified aluminum oxide film against the bacteria of *E. coli* and *S. aureus*. (**e**) Water repellent performance of the surface with the superhydrophobic PFDS/TiO_2_@AAO. (**f**) Schematic illustration of the bacterial adhesion on the surface with the superhydrophobic PFDS/TiO_2_@AAO. (**g**,**h**) Bacterial adhesion testing of the superhydrophobic PFDS/TiO_2_@AAO against the bacteria of *E. coli* and *S. aureus*.

WCA of the samples before and after filled with PFDS/TiO_2_ are tested to investigate the water repellent performance. Contrastively, the WCA changes from 68° (Figure 8a) to 159° (Figure 8e) after the surface is modified with PFDS/TiO_2_, indicating that the water repellent performance transforms from hydrophilic state to superhydrophobic state. Due to the good water repellent property, the superhydrophobic PFDS/TiO_2_@AAO is hardly adhered to by the bacterial colonies of *E. coli* and *S. aureus* (Figure 8g,h). The superhydrophobic PFDS/TiO_2_@AAO performs a 100% inhibition ratio (see calculating details in Experimental Section) against the two bacteria, displaying excellent anti-bioadhesive performance. However, the unmodified aluminum oxide film is seriously adhered to by the bacteria (Figure 8c,d), exhibiting no inhibition effect of bacterial adhesion. These results suggest that the water-repellent property and the anti-bioadhesive performance can be significantly improved by creating thus a superhydrophobic coating on the surface.

**Figure 9:**
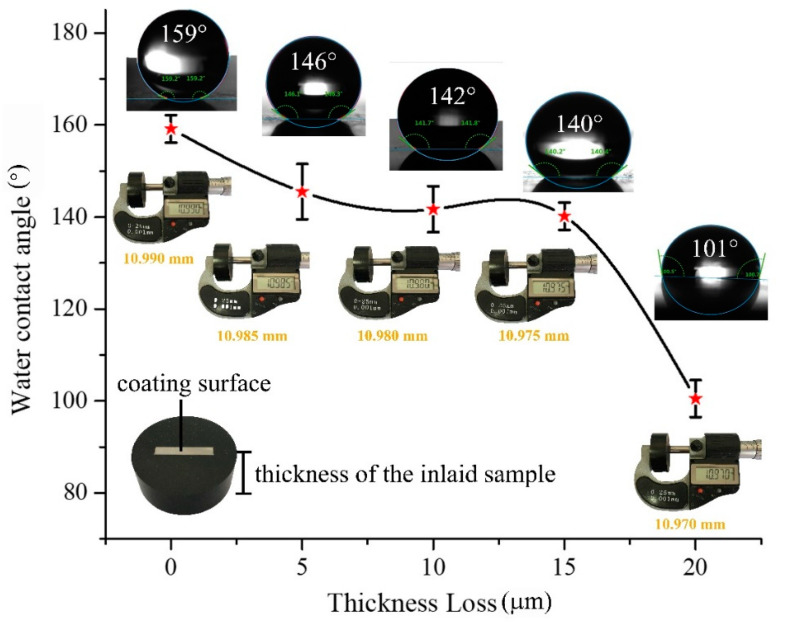
Variation of the WCA with the continuous thickness loss.

**Figure 10:**
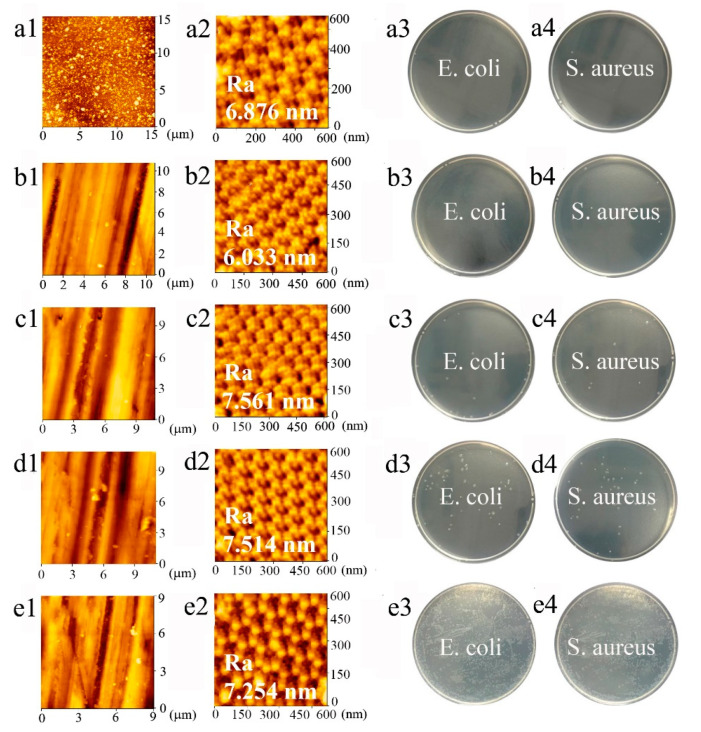
AFM images of the coatings after abrasion and the corresponding anti-adhesion performance at the thickness loss of (**a1**–**a4**) 0 μm, (**b1**–**b4**) 5 μm, (**c1**–**c4**) 10 μm, (**d1**–**d4**) 15 μm, (**e1**–**e4**) 20 μm.

Standard sandpaper abrasion is performed to test the mechanical robustness of the superhydrophobic PFDS/TiO_2_@AAO coating (see the experimental details in the Experimental Section). The WCA changes as functions of the abrasive thickness loss are shown in Figure 9. The WCA remains above 145° after 5 μm thickness loss (Figure 9), revealing that slight abrasion damage has little impact on the coating hydrophobicity. With the increasing thickness loss, the WCA decreases slightly, but still maintains above 140° before a thickness loss of 15 μm. The steep decline in WCA occurs after ~15 μm thickness loss, and the WCA falls to 101° after a total thickness loss of ~20 μm, indicating the failure of the superhydrophobic coating. Here, to keep a consistent surface roughness of the testing samples, all the testing surfaces are kept at the same scale in the microstructures that are characterized by AFM (Figure 10). 

To verify the durability of the anti-bioadhesive performance, bacterial adhesion testing is performed on the superhydrophobic surface before/after abrasion. In the initial state, the surface before abrasion shows good inhibition of bacterial adhesion against *E. coli* and *S. aureus* with no bacterial colony adhesion (Figure 10(a3,a4)). After 5 μm thickness loss, it still displays excellent anti-bioadhesive performance as shown in Figure 10(b3,b4). However, the adhesive bacterial colonies are sporadically distributed on the testing plate after the coating is abraded off 15 μm (Figure 10(d3,d4)). Results in Figure 10(e3,e4) indicate that the coating is entirely ineffective with a mass of bacterial colonies adhered to after ~20 μm thickness loss. These results suggest that the coating can provide effective inhibition of bacterial adhesion within ~15 μm thickness. The robust and durable superhydrophobic surface can be applied in the harsh environment, such as the guild rails of the automobile skylights that serves under the abrasive condition (see the details in Figure S5).

**Figure 11:**
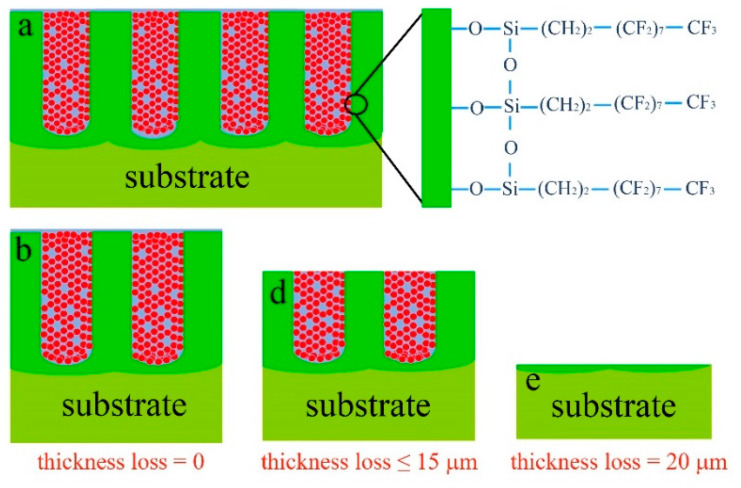
(**a**–**e**) Mechanism schematic of the robust superhydrophobicity against abrasion.

The mechanism schematic in Figure 11 reveals why the PFDS/TiO_2_@AAO is robust against abrasion. As the discussion in Figure 7 and Figure 3c,d, the functional organic groups have been grafted onto the wall of the nanopores, and the nanoparticles-induced modifier is filled deep into the bottom of the nanopores. During the abrasion (Figure 11b,c), the upper layer of the PFDS/TiO_2_@AAO is abraded while the remaining layer can still provide the hydrophobicity benefiting from the self-similar structure of the nanoporous framework. Until the thickness loss achieves to ~20 μm (Figure 11d), the PFDS/TiO_2_@AAO is completely expended during the continuous abrasion, and the bulk substrate material exposes, which fails the superhydrophobicity. 

**Figure 12:**
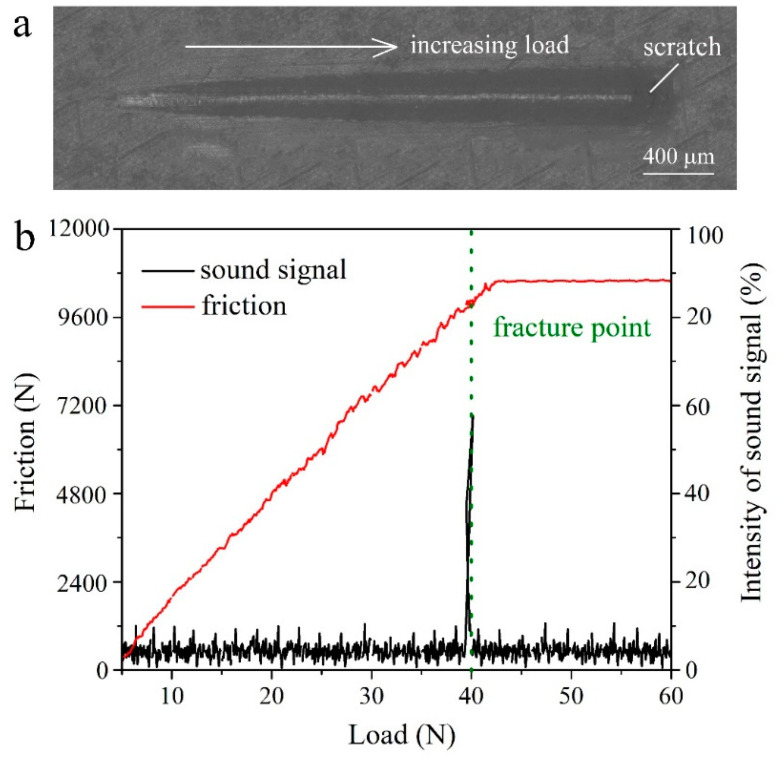
(**a**) Variation curves of the friction and (**b**) the sound signal with the increasing scratch load obtained via the scratch test.

**Figure 13:**
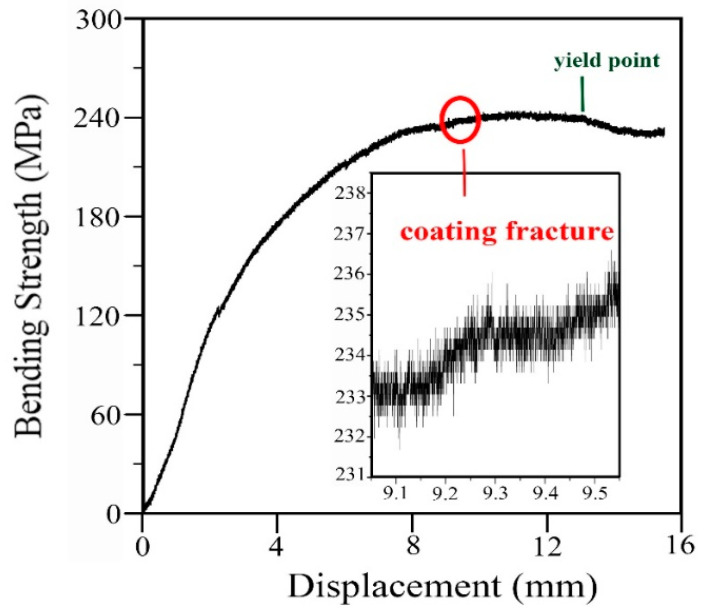
Variation curve of bending strength with the bending displacement achieved via bending test.

**Figure 14:**
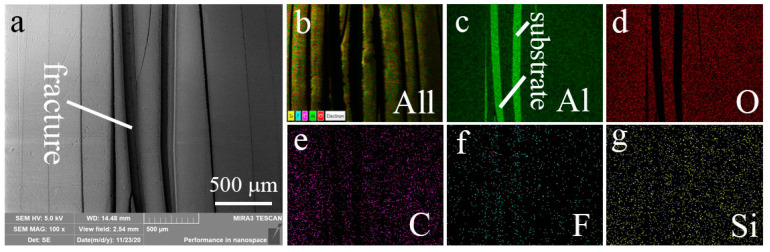
(**a**) SEM image and (**b**–**g**) EDS elemental mapping of the bending fracture.

In practice, adhesion parameters are important to a superhydrophobic surface. To evaluate the adhesion of the coating, scratch test, and bending test are employed in this work. Scratch test can provide the anti-scratch ability of the surface. Figure 12a shows the scratch that broadens and deepens with the increasing load. The variations of friction and sound signal with the increasing loading are recorded by the tester. Figure 12b shows that the friction and sound signal concurrently occur saltation under 40 N load, which indicates the coating fracture. The bending test can directly give the bending strength of the coating. Figure 13 exhibits the variation of the bending strength with the displacement. It can be seen that the curve has saltation at the bending strength of 234 MPa that implies the coating cracks. It can be seen that the cracking strength of the coating is near to the yield strength of the sample (244 MPa), which suggests that the coating has an excellent anti-bending performance. SEM image of the bending sample (Figure 14a) displays that the surface coating has cracked after the bending test. EDS mapping (Figure 14b–g) verify the cracks happen on the total coating because the substrate has been exposed between the cracks. 

## 4. Conclusions

In summary, we design an organic–inorganic superhydrophobic surface with robustness against abrasion by filling the high-hardness nanoporous aluminum oxide film with the low-surface-energy PFDS/TiO_2_. The surface exhibits superhydrophobicity with a large WCA of 159°, and the WCA can keep above 140° even after a ~15 μm thickness loss. Besides, the coating performs durable efficient inhibition of the bacterial adhesion under continuous abrasion. For engineering applications, this superhydrophobic surface has the intrinsic high hardness and strong adhesive force, and it can be achieved by a high-output fabricating method at a low-cost. Furthermore, the design strategy of the robust surface with the special microstructure can be widely adapted to the other materials that need stable and robust armor for service in harsh environments.

## Figures and Tables

**Table 1:** Electrolytes and parameters in the anodizing process.

Electrolyte	Concentration (wt%)	Constant Voltage (V)	Temperature (°C)	Time (min)
H_3_PO_4_	15	40/50/60/70	20	45
H_2_SO_4_	11	20/25/30/35	0	45
OA	5	40/45/50/55	0	45

**Table 2:** Average pore size, porosity, and Vickers’ hardness of the samples (sample no. as same as that in Figure 2).

Sample No.	Average Pore Size (nm)	Porosity (%)	Hardness (HV)
#a1	46.3	61.5	131.7 ± 2.2
#a2	69.1	54.4	109.4 ± 3.0
#a3	96.0	41.9	100.2 ± 2.1
#a4	120.4	47.2	94.3 ± 3.5
#b1	33.1	71.4	271.5 ± 3.9
#b2	55.0	66.3	260.4 ± 5.8
#b3	81.1	61.6	234.1 ± 5.1
#b4	86.7	51.7	205.9 ± 4.6
#c1	37.3	62.1	427.2 ± 4.7
#c2	56.7	59.4	409.8 ± 3.1
#c3	74.2	57.1	394.5 ± 4.0
#c4	100.9	45.9	363.9 ± 3.6

## Supplementary Materials

The following are available online at https://www.mdpi.com/1996-1944/13/23/5564/s1, Figure S1: The schematic illustration of the abrasion experiment; Figure S2: The statistical image of the porosity calculation; Figure S3: The micrograph of the hardness test; Figure S4: The SEM images of the surface modified with different amount of TiO_2_ nanoparticles; Figure S5: The pictures of the guild rails that occur cracks (general anodizing film) and keep complete (robust superhydrophobic film).
