# Supplementary material for: A Stable and Indurative Superhydrophobic Film with Excellent Anti-Bioadhesive Performance for 6061 Al Protection
Source: Materials (Basel). 2020 Dec 6;13(23):5564. doi: 10.3390/ma13235564 (PMC7731204; doi:10.3390/ma13235564)
Supplement: Supplementary file 1 [file materials-13-05564-s001.pdf]

materials

Supporting Information

A Stable and Indurative Superhydrophobic Film with Excellent Anti-Bioadhesive Performance for 6061 Al Protection

Jie Liu [1], Xinwen Zhang [1], Ruoyun Wang [1], Fei Long [1] and Lei Liu [1,2,*]

[1] State Key Laboratory of Metal Matrix Composites, School of Material Science and Engineering, Shanghai Jiao Tong University, Shanghai 200240, China; lindialj@sjtu.edu.cn (J.L.); zhangxw@sjtu.edu.cn (X.Z.); microarc@sjtu.edu.cn (R.W.); longfeimse@sjtu.edu.cn (F.L.)

[2] Collaborative Innovation Center for Advanced Ship and Deep-Sea Exploration, Shanghai Jiao Tong University, Shanghai, 200240, China

* Correspondence: anodic@sjtu.edu.cn; Tel: +86-21-34203812

Received: 5 November 2020; Accepted: 1 December 2020; Published: 6 December 2020

1. Abrasion Experiment

The surface abrasion was conducted via the cyclic linear motion of the emery paper (2000#) under the fixed weight (500 g), and the schematic illustration was shown in Supporting Information (Figure S1). The length of the testing surface is 2 cm. The moving speed of the weight is 0.1 m/s.

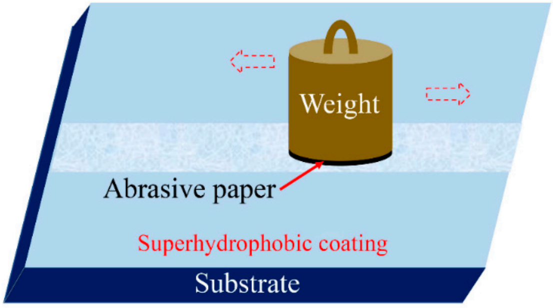

Figure S1. Schematic illustration of the abrasion experiment.

2. Calculation of Porosity

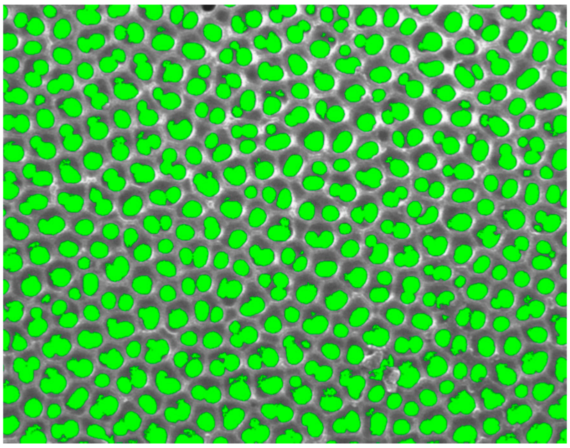

Figure S2. Statistical image of the porosity calculation.

The porosity is calculated from the area in green divided by the total area in the statistical image. The calculated value is as shown in Table 1 in the text.

3. Hardness

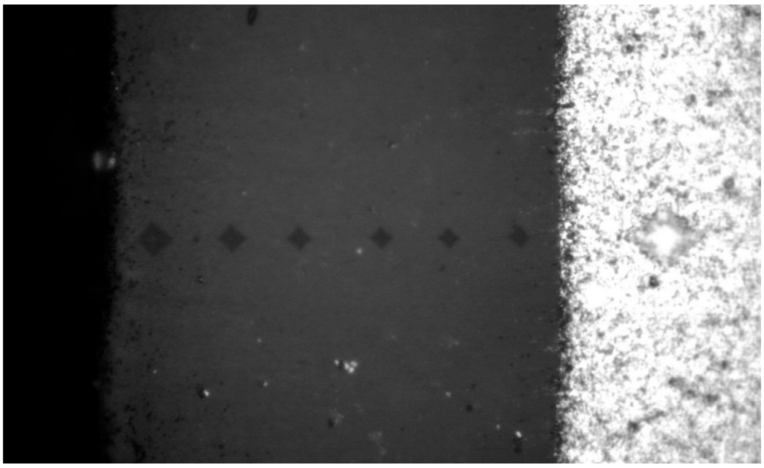

Figure S3. Micrograph of the hardness test.

The hardness is tested by a microhardness tester. The testing image is as shown in Figure S3. The hardness value refers to the hardness of the outer layer, that is to say, the largest one. The hardness values of all the testing samples are as shown in Table 1 in the text.

4. Theoretical WCA Based on the Cassie–Baxter Relation

This coating is composed of a nanoporous inorganic skeleton and an organic hydrophobic modifier. Considering the inevitable existence of air in the pores, it conforms to the characteristics of the Cassie–Baxter hydrophobic model. Therefore, the superhydrophobic surface belongs to the Cassie–Baxter surface. According to the Cassie–Baxter theory, there exists a composite interface composed of liquid–solid and liquid–gas under the droplet, and the Cassie–Baxter equation is proposed as

$$\cos\theta_{\gamma} = f_{ls} \times (\cos\theta_0 + 1) - 1$$

wherein, θ_{γ} is the apparent contact angle, θ_0 is the intrinsic contact angle, and f_{ls} is the percentage of the area occupied by the liquid–solid interface. In the wetting state of Cassie–Baxter, the presence of air in the pore structure reduces the liquid–solid contact area, that is, f_{ls} decreases. Here, the θ_0 is 148°, and the porosity is 57.1%. According to the equation, we can get that the theoretaical WCA is about 160°.

5. Influence of the Amount of TiO_2 Nanoparticles to Microstructures

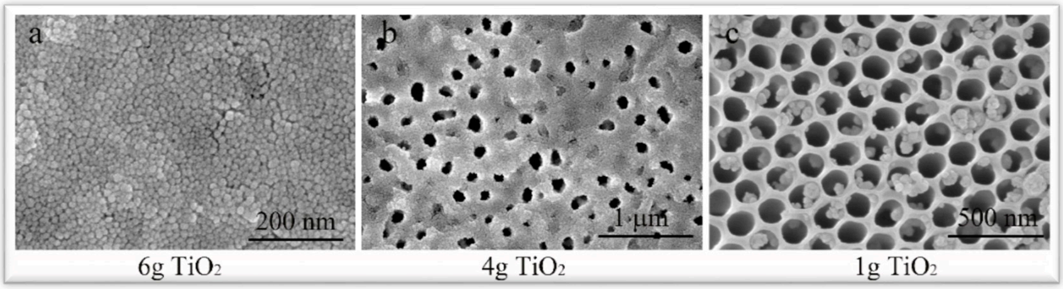

Figure S4. SEM images of the surface modified with different amount of TiO_2 nanoparticles.

The optimized amount of TiO_2 nanoparticles was decided after the testing campaign (see the added SEM image in Figure S4 in the revised Supporting Information). Too much amount of TiO_2 will cause the serious aggregation of nanoparticles and result in the blocking of nanochannels (Figure S4a,b). Too less amount of TiO_2 is not enough to completely fill the nanopores (Figure S4c), and the near empty nanoporous framework can not satisfy the demand of superhydrophobicity and robustness against abrasion. Thus, we choose the optimized amount of TiO_2 (2g/101g, i.e. 19.8 wt%) after experimental studies.

6. Application Example

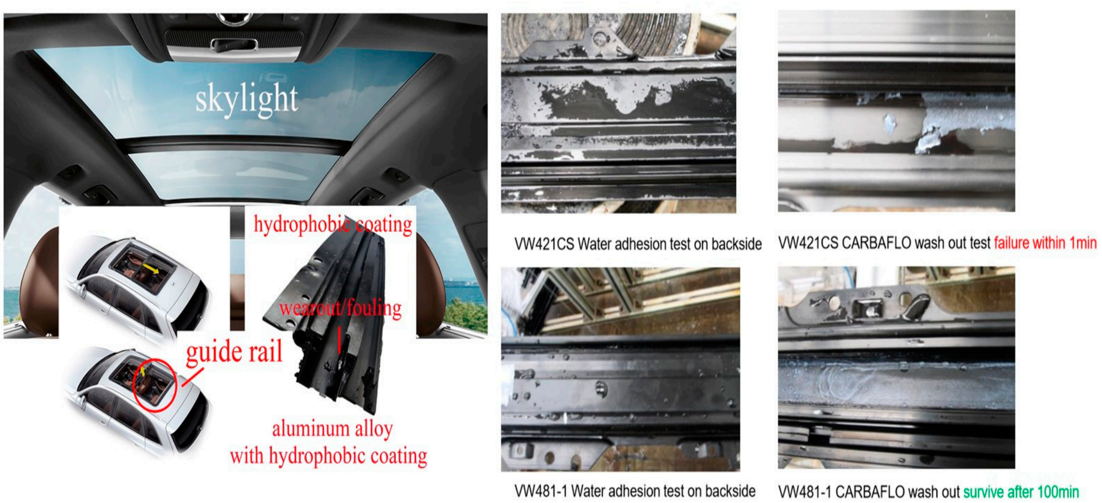

Figure S5. The pictures of the guide rails that crack (general anodizing film) and keep complete (robust superhydrophobic film).

© 2020 by the authors. Licensee MDPI, Basel, Switzerland. This article is an open access article distributed under the terms and conditions of the Creative Commons Attribution (CC BY) license (http://creativecommons.org/licenses/by/4.0/).
